# Effects of the types of medial meniscal tears on the degree and site of meniscal extrusion

**DOI:** 10.1002/jeo2.70686

**Published:** 2026-03-22

**Authors:** Atsushi Ichiba, Junya Matsuyama

**Affiliations:** ^1^ Department of Orthopedic Surgery Shinkawabata Hospital Kyoto Japan

**Keywords:** magnetic resonance imaging, medial meniscus, meniscal extrusion, meniscal tear type, osteoarthritis

## Abstract

**Purpose:**

Medial meniscal (MM) tears and extrusion contribute to knee osteoarthritis (OA) progression. This study investigated the effects of MM tear type on the degree and site of extrusion using magnetic resonance imaging (MRI) to assist in clinical treatment decision‐making.

**Methods:**

We analysed 115 knees (58.3% women; mean age 63 years). OA severity was assessed using Kellgren–Lawrence (K–L) classification: K–L 0–1 (Group E), K–L ≥ 2 (Group A). Extrusion was measured at 13 points from anterior to posterior horn at 15° intervals on MRI. Patients were categorized by MM tear type: no tear (EN), longitudinal (EL), horizontal (EH), complex (EC) and posterior root (ER) in Group E. In Group A, complex (AC) and posterior root (AR) tears were analysed. Data were analysed using analysis of variance (ANOVA).

**Results:**

Extrusion was significantly greater in the middle segments of EH, EC and ER groups than EN, whereas no significant difference was observed in EL. Marked middle segment extrusion was observed in ER. In AC, extrusion was greater than EN from anterior to posterior, whereas in AR, extrusion was significantly greater in middle and posterior segments.

**Conclusion:**

MM extrusion is influenced by the type of meniscal tear and the severity of OA. Complex and posterior root tears result in increased middle to posterior extrusion. Understanding the relationship between the different types of MM tears and extrusion patterns can aid early detection of high‐risk tears and guide treatment strategies to prevent OA progression.

**Level of Evidence:**

Level IV.

**Abbreviations**
BMI: body mass index
FTA: femorotibial angle
ICC: intraclass correlation coefficient
K–L: Kellgren–Lawrence classification
MM: medial meniscus
MME: medial meniscal extrusion
MMPRT: medial meniscus posterior root tear
MRI: magnetic resonance imaging
OA: osteoarthritis

## INTRODUCTION

Several studies have reported that knee osteoarthritis (OA) progression is more pronounced when accompanied by medial meniscal extrusion (MME) [6, 7, 17, 18, 20]. Factors influencing MME include female sex, bone marrow lesions, cartilage damage, high body mass index (BMI), radiographic OA and medial meniscal (MM) tears [10, 31]. Meniscal tears can be classified into several types, including longitudinal, radial, beak, horizontal and flap tears [3]. Degeneration or tearing of the meniscus disrupts its circumferential fibres, leading to extrusion and increased cartilage loading [16]. The specific type of meniscal tear determines the manner and extent of circumferential fibre disruption, which in turn affects the degree of damage to the articular cartilage [13, 21]. Therefore, identifying the types of meniscal tears most strongly associated with extrusion may help guide treatment strategies. However, aside from medial meniscal posterior root tears (MMPRTs) [10, 12, 15], few studies have examined the association between meniscal tear type and MME [9, 27, 30, 31]. Consequently, treatment strategies for MMPRTs have been widely discussed in the literature [8, 11, 29].

Meniscal extrusion is typically measured using a single magnetic resonance imaging (MRI) slice at the midbody of the meniscus [9, 22]. Only a few studies have assessed the site‐specific characteristics of extrusion [14]. A previous study used radial MRI to create 12 radial coronal slices extending from the anterior to the posterior horn of the MM, finding that MME was greater in the anterior horn in non‐ or early‐stage OA [20]. In the study, radial coronal sections of the MM were created to evaluate the degree of MME. Although increased MME was observed in association with MM tears, extrusion was not evaluated according to specific meniscal tear types. To the best of our knowledge, few studies have investigated how different types of MM tears affect both the degree and site of extrusion.

In this study, we aimed to measure the MME across anterior to posterior segments of the MM and to investigate the association between the types of MM tears and the degree and site of extrusion. By clarifying the association between tear morphology and extrusion patterns, this study aims to enhance clinical understanding and support optimization of treatment strategies for meniscal extrusions.

We hypothesized that (1) longitudinal and horizontal MM tears would have minimal effects on the degree and site of extrusion due to limited disruption of circumferential fibres, and (2) MMPRTs would result in greater extrusion, particularly in the posterior segment.

## METHODS

### Study participants

This study was conducted with approval from the local institutional review board from which the data were collected (IRB No. 202401). This retrospective study analysed routinely collected clinical data from patients aged 40 years or older who presented with knee symptoms and underwent unilateral knee radiography and MRI between June 2021 and August 2022. The decision to perform MRI was made by orthopaedic surgeons based on a comprehensive clinical assessment, including patient history and physical examination findings during outpatient visits. Inclusion and exclusion criteria are shown in Table 1.

**Table 1:** Inclusion and exclusion criteria.

Inclusion criteria
Presentation at the orthopaedic outpatient clinic with a chief complaint of knee pain
Underwent radiography and MRI
Body mass index ≦ 30 kg/m^2^
Age ≧ 40 years old
Valgus deformity defined as a femorotibial angle ≥ 170°
Evaluable medial meniscus on MRI
Exclusion criteria
Body mass index > 30 kg/m^2^
Age < 40 years old
Valgus deformity defined as a femorotibial angle < 170°
Fracture around the knee
Dislocation of the knee
Ligament injury of the knee
Previous knee surgery
Difficult to measure the meniscal extrusion
Tumours around the knee

Abbreviation: MRI, magnetic resonance imaging.

### Radiographic assessment

A series of knee radiographs, including standing anteroposterior, lateral and skyline views, was obtained for each patient. The severity of OA was graded using the Kellgren–Lawrence (K–L) classification system [23]. In this study, a K–L grade of ≥2 was defined as radiographic OA. The anatomical femorotibial angle (FTA) was also measured. All radiographic images were digitally acquired using a picture archiving and communication system (PACS) (Rapideye; Toshiba Medical Systems), and all measurements were performed using PACS software.

### MRI assessment

MRI was performed using a 1.5‐T Vantage XGV scanner (Toshiba Medical Systems) equipped with a dedicated knee coil, with the knee positioned in full extension. The standard imaging protocol included the following sequences: proton density‐weighted coronal (TR/TE, 2200/15 ms), sagittal T1‐weighted (TR/TE, 465/12 ms), coronal T2*‐weighted (TR/TE, 530/15 ms), sagittal T2*‐weighted (TR/TE, 530/15 ms) and radial T2*‐weighted (TR/TE, 560/15 ms).

### Measurement of MME in radial sections

Figure 1a illustrates the method used to determine slice positions on MRI [20]. An axial view of the MM was obtained using T2*‐weighted imaging. The centre of the anteroposterior diameter of the anterior horn of the MM was designated as Point A, and the centre of the posterior horn was designated as Point B. A line connecting Points A and B was drawn, and its midpoint was defined as Point M. Twelve radial slices were obtained at 15° intervals around Point M, covering the entire circumference of the MM. The MM was divided into three regions: the anterior horn, meniscal body and posterior horn. Slice Positions 1–4, 5–9 and 10–13 were defined as the anterior, middle and posterior segments, respectively. Slice Position 1 is referred to as P1 in this study. MME was measured in each slice as the distance from the tibial articular surface to the outer edge of the MM, excluding osteophytes (Figure 1b). A vertical reference line was drawn along the outer margin of the medial tibial plateau, and extrusion was measured as the perpendicular distance from this line to the outermost border of the MM.

**Figure 1:**
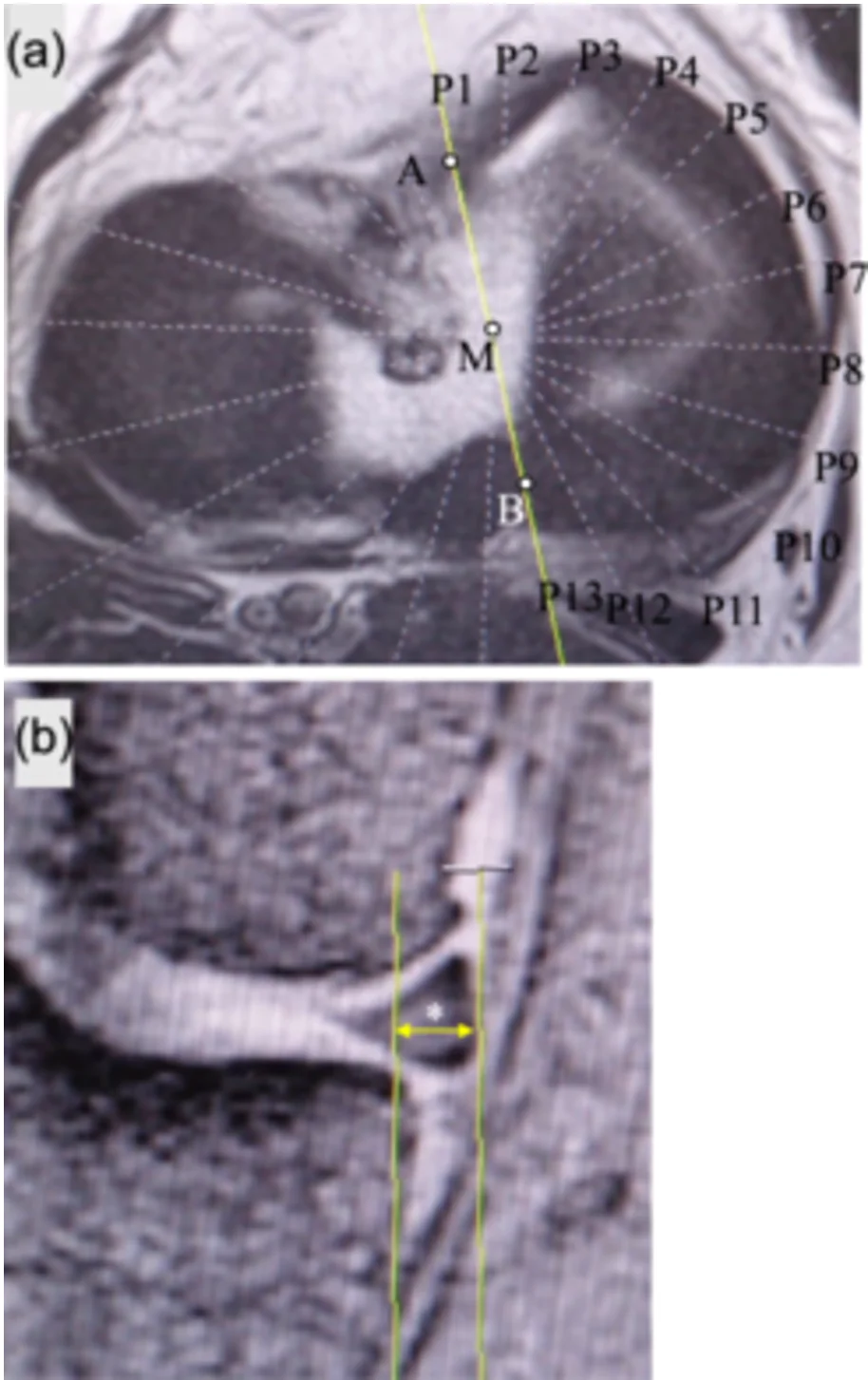
(a) Schematic illustration of slice selection on magnetic resonance imaging (MRI). Point A: Centre of the anteroposterior diameter of the anterior horn of the medial meniscus. Point B: Centre of the anteroposterior diameter of the posterior horn of the medial meniscus. Point M: The midpoint of line AB. The slices passing through point M were designated as P1 to P13. (b) In each slice, medial meniscal extrusion was measured from the tibial articular surface, excluding osteophytes (*). Displacement of the medial meniscus from the margin of the medial tibial plateau was assessed. A vertical reference line was drawn at the outer margin of the medial tibial plateau, and extrusion was defined as the distance from this line to the outer edge of the medial meniscus.

### Classification of meniscal tears

The types of MM tears were evaluated using axial, coronal and sagittal MRI sections. Meniscal tears were classified according to the criteria established by the International Society of Arthroscopy, Knee Surgery, and Orthopaedic Sports Medicine (ISAKOS) [3]. In this study, MM tears were categorized into four types: longitudinal, horizontal, complex and MMPRT. Horizontal tears were defined as cleavage lesions parallel to the tibial plateau, resulting in a horizontally split meniscus. Radial tears excluding MMPRTs, as well as oblique and flap tears, were classified as complex tears based on disruption of circumferential fibres in order to simplify the data analysis. A meniscal tear was defined as a linear intrameniscal signal extending to at least one articular surface. An MMPRT was defined as a radial tear located within 9 mm of the posterior bony attachment of the MM [26] and diagnosed based on previously reported criteria [19]. MRI assessments were conducted independently of clinical data and OA grading.

### Subgroup analysis

Patients with a K–L grade of 0 or 1 were classified into Group E (early), whereas those with K–L grades 2, 3 or 4 were classified as Group A (advanced). Subgroup analyses were performed to investigate the association between the types of MM tears and MME. In Group E, cases without MM tears were designated as Group EN; those with longitudinal tears as Group EL; those with horizontal tears as Group EH; those with complex tears as Group EC; and those with MMPRTs as Group ER. In Group A, cases without MM tears were designated as Group AN; those with longitudinal tears as Group AL; those with horizontal tears as Group AH; those with complex tears as Group AC; and those with MMPRTs as Group AR.

### Evaluations

First, the degree of MME was measured at 13 slice positions obtained from the radial MRI scans (P1–P13). Second, the maximum extrusion value in each knee was identified and defined as the site of maximum extrusion. Third, the relationships between the types of MM tears and both the degree and site of MME were analysed.

### Reproducibility measurements

The reliability of the assessment methodology used in this study has been reported previously [20]. Inter‐observer reliability for the K–L grading demonstrated a *κ* coefficient of 0.93. The reproducibility of MME measurements was assessed using the intraclass correlation coefficient (ICC), with intraobserver and interobserver ICCs of 0.90 and 0.88, respectively. Inter‐observer reliability for the classification of MM tear types showed a *κ* coefficient of 0.82. Meniscal tear types were independently diagnosed by two experienced orthopaedic surgeons.

### Statistical analysis

Data are presented as mean ± standard deviation. Comparisons between two groups were conducted using Student's *t* test. Demographic variables and MME values were compared using one‐way analysis of variance (ANOVA) followed by Tukey's post hoc test. The distribution of the site of maximum extrusion among groups was compared using the chi‐squared test. A *p* value of <0.05 is considered statistically significant. All statistical analyses were performed using Bell Curve for Excel (Social Survey Research Information Co. Ltd.). Prior to the study, a sample size calculation focusing on MME was conducted using G*Power 3.0 (Heinrich‐Heine‐Universität). To achieve 80% statistical power with an alpha level of 0.05 and a large effect size (*f* = 0.4), the analysis indicated that a minimum total sample size of 98 patients was required to detect significant differences in MME using ANOVA.

## RESULTS

### Demographics

A total of 115 knees (115 patients) met the inclusion criteria and were included in the final study cohort (Figure 2). Among these patients, 58.3% were women. The mean age was 63.6 ± 11.2 years, and the mean BMI was 23.0 ± 2.9 kg/m^2^ (Table 2). Demographic characteristics of the subgroups are summarized in Table 2. The mean age of Group EN was significantly lower than that of Groups EC, ER and AC. The FTA was significantly greater in Group AR than in Groups EH and AC. Owing to the small number of cases (two in Group AN, one in Group AL and two in Group AH), these subgroups were excluded from the statistical analysis.

**Figure 2:**
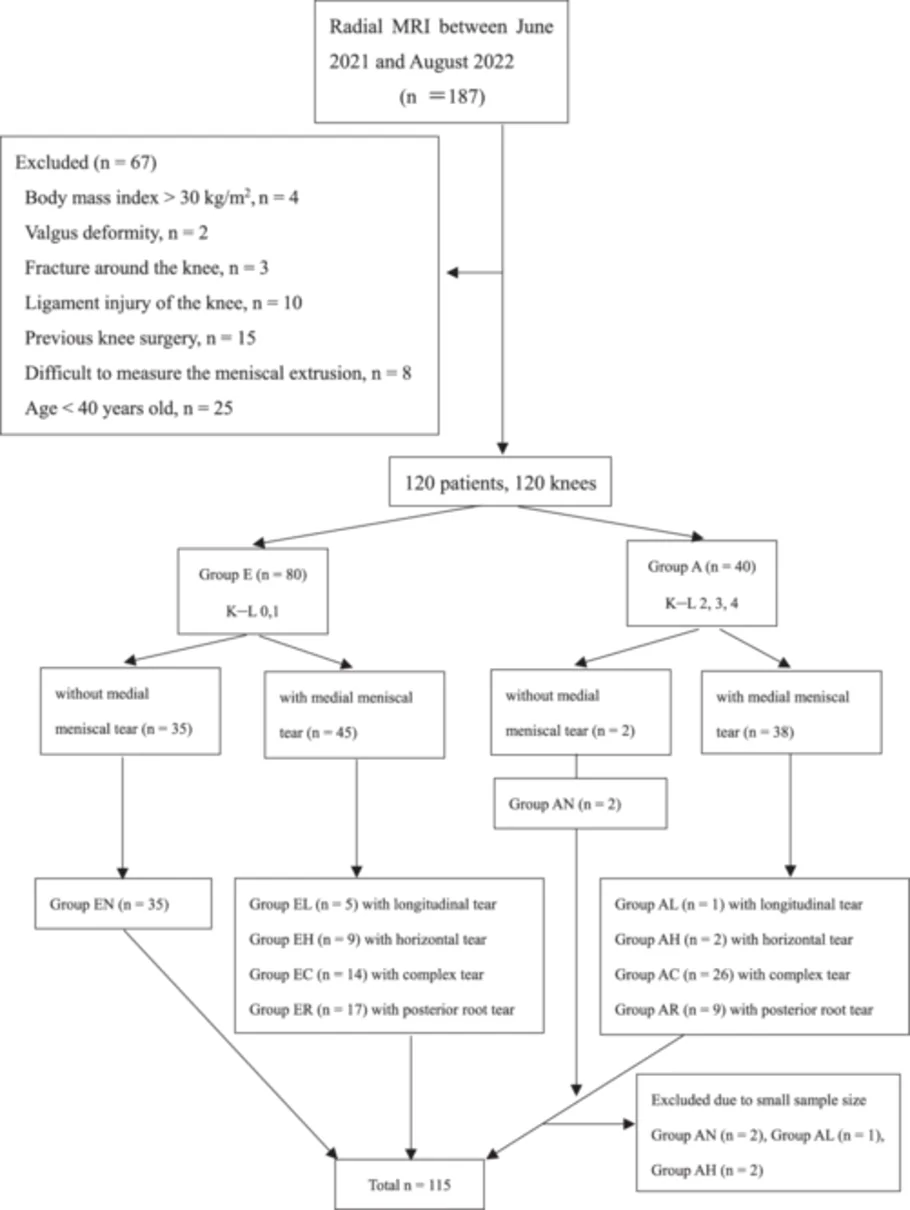
Flowchart summarizing the patient inclusion and exclusion process. K–L, Kellgren–Lawrence grade; MM, medial meniscus; MRI, magnetic resonance imaging; *n*, number. Group definitions: Group E, non‐ or early osteoarthritis (Kellgren–Lawrence Grade 0 or 1); Group EN, non‐ or early osteoarthritis without meniscal tear; Group EL, non‐ or early osteoarthritis with longitudinal medial meniscal tear; Group EH, non‐ or early osteoarthritis with horizontal medial meniscal tear; Group EC, non‐ or early osteoarthritis with complex medial meniscal tear, and Group ER, non‐ or early osteoarthritis with posterior root tear of the medial meniscus. Group A, advanced osteoarthritis (Kellgren–Lawrence Grade ≥ 2); Group AH, advanced osteoarthritis with horizontal medial meniscus tear; Group AC, advanced osteoarthritis with complex medial meniscal tear; Group AL, advanced osteoarthritis with longitudinal medial meniscal tear; Group AN, advanced osteoarthritis without medial meniscal tear and Group AR, advanced osteoarthritis with posterior root tear of the medial meniscus.

**Table 2:** Demographic data of participants.

	Group E					Group A		*p* value
	EN	EL	EH	EC	ER	AC	AR	
No. of patient/knees	35/35	5/5	9/9	14/14	17/17	26/26	9/9	
Sex, men/women, *n*	17/18	2/3	6/3	6/8	4/13	11/15	2/7	0.16
Age, years (SD), range	56.3 (11.7)	60.8 (10.4)	61.1 (13.8)	66.4 (10.8)	69.5 (9.4)	68.9 (9.0)	64.7 (10.4)	a
	41–80	49–72	40–84	43–82	43–79	54–80	52–79	
BMI, kg/m^2^, (SD), range	22.5 (3.3)	20.6 (1.5)	23.3 (2.0)	23.5 (3.4)	24.0 (3.3)	23.4 (2.6)	22.4 (1.7)	0.25
	16.9–29.1	18.3–22.3	20.5–26.1	17.7–29.0	18.3–29.1	19.1–28.7	20.5–24.8	
Side, right/left, *n*	19/16	3/2	4/5	5/9	5/12	12/14	6/3	0.57
K–L grade Grade 0/1/2/3/4, *n*	22/13/0/0/0	5/0/0/0/0	5/4/0/0/0	6/8/0/0/0	13/4/0/0/0	0/0/5/11/10	0/0/1/7/1	
Femorotibial angle (°) (SD), range	178.3 (2.5)	177.9 (2.2)	176.1 (2.9)	176.1 (1.7)	177.7 (2.3)	179.2 (3.1)	180.9 (5.1)	b
	174.9–188.5	175.0–181.2	173.0–178.0	170.1–180.2	174.1–180.2	174.0–184.9	177.0–193.9	

Abbreviations: BMI, body mass index; Group A, advanced osteoarthritis group; Group AC, advanced osteoarthritis with complex medial meniscal tear; Group AR, advanced osteoarthritis with posterior root tear in the medial meniscus; Group E, non‐ or early osteoarthritis group; Group EC, non‐ or early osteoarthritis with complex medial meniscal tear; Group EH, non‐ or early osteoarthritis with horizontal medial meniscal tear; Group EL, non‐ or early osteoarthritis with longitudinal medial meniscal tear; Group ER, non‐ or early osteoarthritis with posterior root tear in the medial meniscus; K–L grade, Kellgren–Lawrence grade; *n*, numbers; SD, standard deviation; °, degrees.

^a^ Results of statistical analysis: EN versus EC, *p* = 0.024; EN versus ER, *p* = 0.0010; EN versus AC, *p* < 0.001.

^b^ Results of statistical analysis: EH versus AR, *p* = 0.011; EC versus AC, *p* = 0.019; EC versus AR, *p* = 0.0019.

#### Results of MRI assessments

##### MME in all patients

Figure 3 illustrates the distribution of MME across all groups. Overall, extrusion was minimal in Group EN. Group A exhibited greater overall extrusion than Group E. In particular, Group AR demonstrated increased MME in the middle to posterior segments (Table 3).

**Figure 3:**
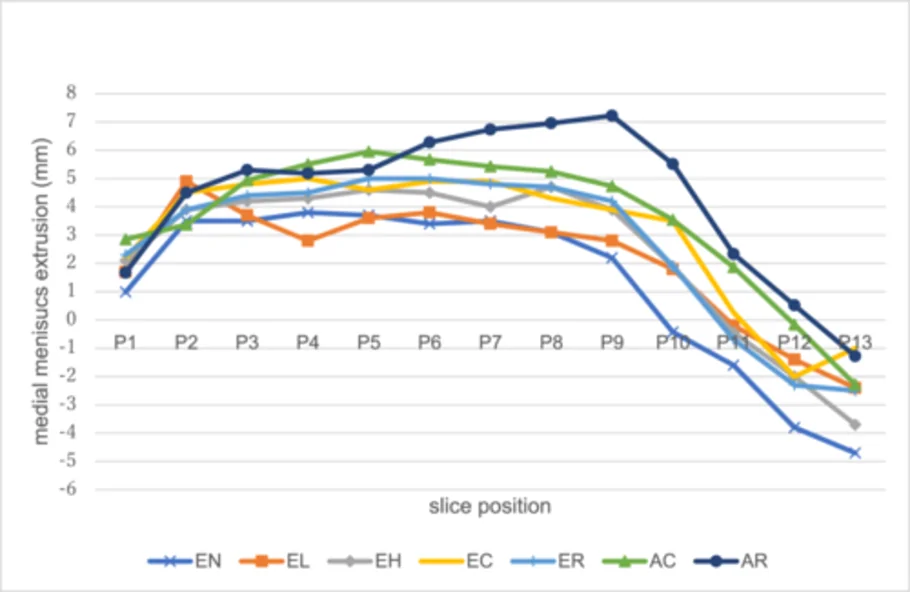
Medial meniscus extrusion in the subgroups. Group AC, advanced osteoarthritis (Kellgren–Lawrence Grade ≥ 2) with complex medial meniscus tear; Group AR, advanced osteoarthritis with posterior root tear of medial meniscus; Group EC, non‐ or early osteoarthritis with complex medial meniscus tear; Group EH, non‐ or early osteoarthritis with horizontal medial meniscus tear; Group EL, non‐ or early osteoarthritis with longitudinal medial meniscus tear; Group EN, non‐ or early osteoarthritis (Kellgren–Lawrence Grade 0 or 1) without medial meniscus tear; Group ER, non‐ or early osteoarthritis with posterior root tear of medial meniscus; P1–P13 refer to coronal slices made radially form the anterior to posterior horn of the medial meniscus on radial MRI, with P1 corresponding to the anterior horn and P13 to the posterior horn. MRI, magnetic resonance imaging.

**Table 3:** Medial meniscal extrusion in subgroups.

	Anterior segment				Middle segment					Posterior segment			
	P1^a^	P2	P3	P4	P5	P6	P7	P8	P9	P10	P11	P12	P13
Group EN													
Mean	0.99	3.48	3.51	3.80	3.71	3.43	3.52	3.11	2.23	−0.43	−1.59	−3.83	−4.56
SD	1.94	2.17	1.44	1.35	1.46	1.14	0.95	1.39	1.59	2.07	2.77	2.60	2.87
Group EL													
Mean	1.72	4.91	3.75	2.82	3.56	3.82	3.44	3.14	2.82	1.82	−0.21	−1.42	−2.40
SD	1.64	2.25	2.22	1.64	0.92	0.73	0.71	0.51	1.58	3.03	2.21	1.94	2.19
Group EH													
Mean	2.11	3.90	4.22	4.33	4.57	4.52	3.96	4.67	3.90	1.94	−0.44	−1.99	−3.68
SD	3.33	1.86	1.75	1.19	1.43	0.79	1.71	0.87	1.07	2.40	3.46	2.80	3.23
Group EC													
Mean	2.19	4.72	4.79	4.99	4.64	4.87	4.87	4.24	3.89	3.54	0.26	−1.96	−4.01
SD	2.54	1.86	1.61	1.21	1.46	1.69	1.26	1.65	1.75	1.79	3.33	3.53	3.03
Group ER													
Mean	2.27	3.90	4.36	4.51	5.00	5.04	4.79	4.72	4.22	1.90	−0.69	−2.33	−2.46
SD	1.86	1.81	1.72	1.76	1.40	1.61	1.60	1.92	1.77	3.63	3.98	2.92	2.43
Group AC													
Mean	2.58	3.37	4.94	5.51	5.95	5.67	5.43	5.25	4.73	3.54	1.87	−0.15	−2.27
SD	2.07	2.39	2.13	1.59	2.42	1.48	1.74	2.24	2.53	3.27	3.39	4.11	3.57
Group AR													
Mean	1.67	4.50	5.31	5.18	5.33	6.28	6.73	6.96	7.22	5.11	2.33	0.52	−1.28
SD	2.53	2.59	1.43	1.95	2.18	2.51	3.03	3.52	3.82	3.69	4.21	4.14	4.13

Abbreviations: Group AC, advanced osteoarthritis with complex medial meniscal tear; Group AR, advanced osteoarthritis with posterior root tear in the medial meniscus; Group EC, non‐ or early osteoarthritis with complex medial meniscal tear; Group EH, non‐ or early osteoarthritis with horizontal medial meniscal tear; Group EL, non‐ or early osteoarthritis with longitudinal medial meniscal tear; Group ER, non‐ or early osteoarthritis with posterior root tear in the medial meniscus; SD, standard deviation.

^a^ P1, coronal slices were made radially from the anterior to posterior horns of the medial meniscus on radial magnetic resonance imaging, with the anterior horn slice as Position 1 and posterior horn slice as Position 13.

##### Association between the types of MM tears and extrusion

Comparison of MME across different types of MM tears is summarized in Table 4. Comparisons between Groups EN and EL, and between EN and EH indicated that longitudinal tears were not associated with significant increases in extrusion at any slice position, whereas horizontal tears showed significantly greater extrusion at P8 and P9 compared with Group EN. Group AC showed significantly greater extrusion across most regions, extending from the anterior to the posterior segments. Comparison between Groups EN and EC demonstrated significantly greater extrusion in Group EC from P6 to P10. Group ER exhibited increased extrusion from P5 to P9 compared with Group EN.

**Table 4:** Results of comparisons between the groups (*p* value and 95% confidence interval).

	P1^a^	P2	P3	P4	P5	P6	P7	P8	P9	P10	P11	P12	P13
EN versus EL	n.s.	n.s.	n.s.	n.s.	n.s.	n.s.	n.s.	n.s.	n.s.	n.s.	n.s.	n.s.	n.s.
EN versus EH 95% CI	n.s.	n.s.	n.s.	n.s.	n.s.	n.s.	n.s.	**0.045**	**0.047**	n.s.	n.s.	n.s.	n.s.
								−2.65	−0.06				
								−0.46	3.38				
EN versus EC 95% CI	n.s.	n.s.	n.s.	n.s.	n.s.	**0.0064**	**0.0062**	n.s.	**0.0013**	**0.0020**	n.s.	n.s.	n.s.
						0.63	−2.11		0.20	0.79			
						2.26	−0.59		3.11	5.35			
EN versus ER 95% CI	n.s.	n.s.	n.s.	n.s.	**0.021**	**<0.001**	**0.0057**	**0.0039**	**<0.001**	n.s.	n.s.	n.s.	n.s.
					0.46	0.85	−1.98	−2.47	0.63				
					2.17	2.37	−0.56	−0.74	3.35				
EL versus EH 95% CI	n.s.	n.s.	n.s.	n.s.	n.s.	n.s.	n.s.	n.s.	n.s.	n.s.	n.s.	n.s.	n.s.
EL versus EC 95% CI	n.s.	n.s.	n.s.	n.s.	n.s.	n.s.	**0.042**	n.s.	n.s.	n.s.	n.s.	n.s.	n.s.
							−2.42						
							−0.44						
EL versus ER 95% CI	n.s.	n.s.	n.s.	n.s.	n.s.	n.s.	**0.048**	n.s.	n.s.	n.s.	n.s.	n.s.	n.s.
							−2.31						
							−0.40						
EH versus EC	n.s.	n.s.	n.s.	n.s.	n.s.	n.s.	n.s.	n.s.	n.s.	n.s.	n.s.	n.s.	n.s.
EH versus ER	n.s.	n.s.	n.s.	n.s.	n.s.	n.s.	n.s.	n.s.	n.s.	n.s.	n.s.	n.s.	n.s.
EC versus ER	n.s.	n.s.	n.s.	n.s.	n.s.	n.s.	n.s.	n.s.	n.s.	n.s.	n.s.	n.s.	n.s.
EN versus AC 95% CI	**0.020**	n.s.	**0.025**	**<0.001**	**<0.001**	**<0.001**	**<0.001**	**<0.001**	**<0.001**	**<0.001**	**<0.001**	**<0.001**	n.s.
	0.74		0.55	1.03	−3.13	1.46	1.13	1.16	1.43	1.64	1.77	2.01	
	2.98		2.30	2.62	−1.34	2.99	2.70	3.10	3.56	4.50	5.16	5.34	
EN versus AR 95% CI	n.s.	n.s.	n.s.	n.s.	n.s.	**<0.001**	**<0.001**	**<0.001**	**<0.001**	**<0.001**	**0.0028**	**0.0082**	n.s.
						1.76	2.08	2.44	3.45	2.97	1.47	1.95	
						3.94	4.34	5.24	6.52	7.10	6.37	6.76	
ER versus AR 95% CI	n.s.	n.s.	n.s.	n.s.	n.s.	n.s.	**0.038**	n.s.	**0.010**	**0.032**	n.s.	n.s.	n.s.
							0.69		1.31	1.32			
							3.19		4.69	5.88			
AC versus AR 95% CI	n.s.	n.s.	n.s.	n.s.	n.s.	n.s.	n.s.	n.s.	**0.033**	n.s.	n.s.	n.s.	n.s.
									0.91				
									4.08				

*Note*: Boldface text indicates statistical significance.

Abbreviations: 95% CI, 95% confidence interval; Group AC, advanced osteoarthritis with complex medial meniscal tear; Group AR, advanced osteoarthritis with posterior root tear in medial meniscus; Group EC, non‐ or early osteoarthritis with complex medial meniscal tear; Group EH, non‐ or early osteoarthritis with horizontal medial meniscal tear; Group EL, non‐ or early osteoarthritis with longitudinal medial meniscal tear; Group ER, non‐ or early osteoarthritis with posterior root tear in medial meniscus; n.s., not significant.

^a^ P1, coronal slices were made radially from the anterior to posterior horns of the medial meniscus on radial magnetic resonance imaging, with the anterior horn slice as Position 1 and the posterior horn slice as Position 13.

Group AC showed significantly greater extrusion across most regions, extending from the anterior to the posterior segments. Similarly, Groups AR demonstrated increased extrusion from P6 to P12 relative to Group EN.

In this study, radial, oblique and flap tears were categorized as complex tears. The detailed distribution of types of MM tear subtypes within the complex tear group is provided in Supporting Information S1: Data S1. Importantly, no cases were identified in which these tear subtypes occurred in isolation. Across the series of one‐way ANOVA analyses conducted at slice positions (P1–P13), group effects were consistently observed, with *F* values ranging from 0.76 to 8.43. Statistically significant differences were identified in the majority of analyses (P3–P12; *p* < 0.05), whereas a small number of comparisons (P1, P2 and P13) did not reach statistical significance. Effect size estimates demonstrated a coherent pattern, with *η* ^2^ values ranging from 0.04 to 0.32. Analyses yielding statistically significant results were predominantly associated with large effect sizes (*η* ^2^ ≈ 0.17–0.32), indicating that approximately 17%–32% of the total variance was explained by group differences. Even in non‐significant analyses, small to moderate effect sizes were observed, suggesting potentially meaningful group‐ related variance (Supporting Information S1: Data S2). Accordingly, interpretation of the results was primarily based on effect size estimates rather than statistical power.

## SITE OF MAXIMUM MME

The distribution of the sites of maximum MME is shown in Figure 4. In Groups EN, EL, EH and EC, maximum extrusion was predominantly observed in the anterior segment. In contrast, in Groups ER, AC and AR, maximum extrusion was more frequently located in the middle segment, with an increasing proportion of cases involving the posterior segment. This distribution differed significantly among the groups (*χ* ^2^ test, *p* = 0.036).

**Figure 4:**
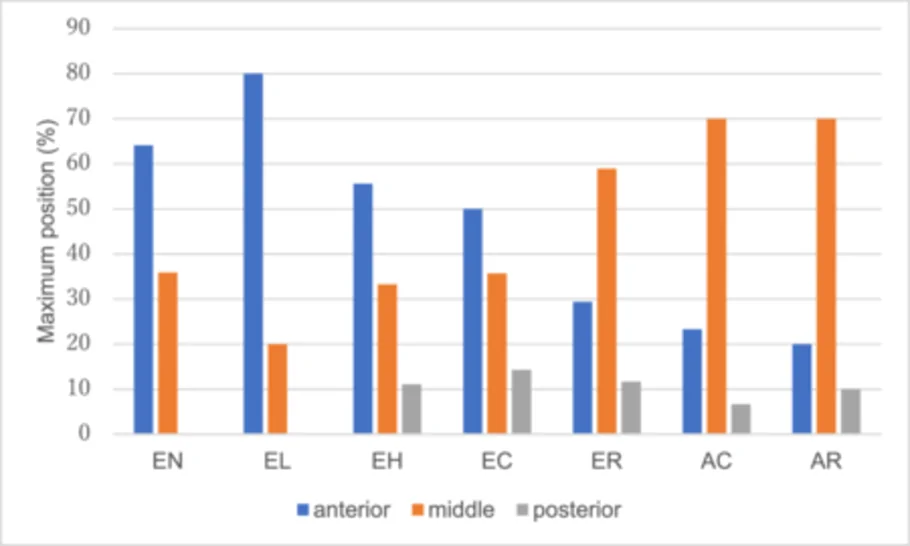
Maximum site of medial mensicus extrusion in each subgroup. Group AC, advanced osteoarthritis (Kellgren–Lawrence Grade ≥ 2) with complex medial meniscus tear; Group AR, advanced osteoarthritis with posterior root tear of medial meniscus; Group EC, non‐ or early osteoarthritis with complex medial meniscus tear; Group EH, non‐ or early osteoarthritis with horizontal medial meniscus tear; Group EL, non‐ or early osteoarthritis with longitudinal medial meniscus tear; Group EN, non‐ or early osteoarthritis (Kellgren–Lawrence Grade 0 or 1) without medial meniscus tear; Group ER, non‐ or early osteoarthritis with posterior root tear of medial meniscus. Segment location definitions: anterior, anterior segment of the medial meniscus; middle, middle segment of the medial meniscus; posterior, posterior segment of the medial meniscus.

## DISCUSSION

The most important finding of this study is that both the degree and site of the MME vary according to the type of MM tear and the stage of OA progression. To the best of our knowledge, this is the first study to clarify the association between the types of MM tears and both the degree and sites of MME.

The first hypothesis was that longitudinal and horizontal tears do not substantially affect meniscal extrusion, which was partially confirmed. Longitudinal tears were not associated with increased extrusion at any slice position. In contrast, horizontal tears were associated with increased extrusion at P8 and P9. The second hypothesis is that MMPRTs would result in greater extrusion, particularly in the posterior segment, which was largely supported. In Group ER, extrusion was significantly increased in the middle segment (P5–9), although no significant increase was observed in the posterior segment. However, analysis of the maximum site of extrusion revealed that Group ER exhibited increased extrusion in both the middle and posterior segments compared with other groups in Group E (Figure 4), indicating a greater posterior impact. In Group AR, extrusion was significantly increased across the middle and posterior segments (P6–12), further supporting the second hypothesis.

Several studies have previously investigated the association between the types of meniscal tears and MME [9, 10, 27, 30, 31]. Zhan et al. reported that, among the various types of meniscal tears, radial tears (odds ratio [OR] = 2.64) and MMPRTs (OR = 8.39) were significantly associated with increased extrusion, whereas horizontal, longitudinal and oblique tears were not [31]. Although Zhan et al. did not evaluate site‐specific extrusion, their findings regarding the association between longitudinal tears, MMPRTs and extrusion are consistent with our results [31]. However, in contrast to their findings, we observed that horizontal tears may contribute to extrusion, specifically in Group EH, which exhibited significantly greater extrusion than Group EN from P8 to P10, particularly in the posterior region of the middle segment (Table 4).

Liu et al. investigated factors associated with MME in patients without OA by comparing cases with and without extrusion ≥3 mm [27]. They reported that radial tears, including MMPRTs (OR = 4.34) and complex tears (OR = 3.07), were associated with increased extrusion, whereas longitudinal and horizontal tears were not. In our study, Group EC exhibited significantly greater extrusion than Group EN from P6 to P10. Although Liu et al. classified MMPRT as radial tears, unlike our classification method, their findings were generally consistent with ours. However, a key difference between the two studies concerns the impact of horizontal tears on MME. One possible explanation is methodological; Liu et al. measured extrusion using a single mid‐coronal MRI slice, whereas our study assessed extrusion circumferentially from the anterior to posterior segments. This comprehensive approach may account for the observed differences in the influence of horizontal tears.

With respect to horizontal tears, Arno et al. reported that horizontal tears in the posterior horn increased peak contact pressure by 13% and reduced contact area by 6%, thereby contributing to OA progression [4]. These findings align with our results, indicating that horizontal tears can contribute to meniscal extrusion. Complex tears, which involve substantial disruption of circumferential fibres, are expected to result in greater meniscal extrusion. In our study, Group AC exhibited a significant increase in extrusion across the anterior, middle and posterior segments (Table 4), supporting this rationale.

Numerous studies have investigated the relationship between MMPRTs and increased MME [12, 15, 25, 31]. Injury to the posterior root disrupts hoop stress transmission, resulting in altered knee biomechanics and kinematics [2]. These findings are consistent with the results of our study.

This study has several limitations. First, its retrospective design may introduce selection bias. Second, arthroscopic confirmation of meniscal tear types was not performed; however, MRI has been shown to have a high diagnostic accuracy for meniscal tears [24]. Third, the influence of tear size and location within the MM was not evaluated, despite their known impact on extrusion [5]. Fourth, all MRI examinations were performed under non‐weight‐bearing conditions, which may differ from weight‐bearing imaging in the assessment of MME [1]. Finally, MRI scans were obtained with the knee in near extension. Previous work by Matsuda et al. demonstrated increased posterior extrusion in the 90° flex position using open MRI [28]. Therefore, posterior extrusion may have been underestimated in our study due to anterior displacement of the meniscus in the extended position.

## CONCLUSION

This study highlights the relationship between the types of MM tears and MME. In Group E, extrusion increased in the presence of complex tears and MMPRTs, predominantly in the middle segments. Horizontal tears were also associated with increased extrusion. In Group A, complex tears led to increased extrusion across all segments, whereas MMPRTs mainly affected the middle to posterior segments. These findings emphasize the impact of MM tear types and OA progression on MME. Recognition of specific tear patterns associated with significant extrusion may help in refining treatment strategies, improving patient outcomes and potentially delaying OA progression.

## AUTHOR CONTRIBUTIONS

**Atsushi Ichiba** and **Junya Matsuyama**: Concept and design of study; conducting study and extracting data; interpreting and analysing data; writing and editing manuscript; approving final version of manuscript.

## CONFLICT OF INTEREST STATEMENT

The authors declare no conflicts of interest.

## ETHICS STATEMENT

This study was carried out with local ethical approval from the institution from which data was collected (IRB number: 202401). Informed consent was obtained from all individual participants included in the study.

## Supporting information

Supple_data.

## Data Availability

The datasets generated and/or analysed during the current study are not publicly available due to ethical and privacy restrictions, but are available from the corresponding author on reasonable request.
