# Supplementary material for: Effects of the types of medial meniscal tears on the degree and site of meniscal extrusion
Source: J Exp Orthop. 2026 Mar 22;13(1):e70686. doi: 10.1002/jeo2.70686 (PMC13679886; doi:10.1002/jeo2.70686)
Supplement: Supplementary file 1 [file JEO2-13-e70686-s001.docx]

|  | **F** | **η²** | **Cohen's f** |
| --- | --- | --- | --- |
| P1 | 1.95 | 0.098 | 0.33 |
| P2 | 0.76 | 0.041 | 0.21 |
| P3 | 2.57 | 0.125 | 0.38 |
| P4 | 4.78 | 0.21 | 0.52 |
| P5 | 4.61 | 0.204 | 0.51 |
| P6 | 8.19 | 0.315 | 0.68 |
| P7 | 8.28 | 0.305 | 0.66 |
| P8 | 6.69 | 0.271 | 0.31 |
| P9 | 8.43 | 0.319 | 0.68 |
| P10 | 5.8 | 0.244 | 0.57 |
| P11 | 3.58 | 0.166 | 0.45 |
| P12 | 4.11 | 0.186 | 0.48 |
| P13 | 2.05 | 0.102 | 0.34 |

**Supplemental data 1**

**Results of analysis of variance (ANOVA)**

**Supplemental data 2**

**Classification of types of meniscal tears diagnosed as complex tears**

**Group EC (n = 14)**
Horizontal + massive: 7
Oblique + longitudinal: 1
Oblique + horizontal: 2
Horizontal + longitudinal: 1
Horizontal + radial: 2
Longitudinal + massive: 1

**Group AC (n = 26)**
Horizontal + longitudinal: 14
Horizontal + longitudinal + flap: 1
Horizontal + longitudinal + oblique: 1
Horizontal + radial: 3
Horizontal + flap: 1
Horizontal + beak: 1
Massive: 1
Longitudinal + flap: 1
Longitudinal + radial: 2
Horizontal + massive: 1
